# Application of PTR-MS for Measuring Odorant Emissions from Soil Application of Manure Slurry

**DOI:** 10.3390/s150101148

**Published:** 2015-01-09

**Authors:** Anders Feilberg, Pernille Bildsoe, Tavs Nyord

**Affiliations:** Department of Engineering, Aarhus University, Hangøvej 2, DK-8200 Aarhus N, Denmark; E-Mails: pernille.bildsoe@eng.au.dk (P.B.); tavs.nyord@eng.au.dk (T.N.)

**Keywords:** slurry application, proton-transfer-reaction mass spectrometry, ozonation, separation, odor emission

## Abstract

Odorous volatile organic compounds (VOC) and hydrogen sulfide (H_2_S) are emitted together with ammonia (NH_3_) from manure slurry applied as a fertilizer, but little is known about the composition and temporal variation of the emissions. In this work, a laboratory method based on dynamic flux chambers packed with soil has been used to measure emissions from untreated pig slurry and slurry treated by solid-liquid separation and ozonation. Proton-transfer-reaction mass spectrometry (PTR-MS) was used to provide time resolved data for a range of VOC, NH_3_ and H_2_S. VOC included organic sulfur compounds, carboxylic acids, phenols, indoles, alcohols, ketones and aldehydes. H_2_S emission was remarkably observed to take place only in the initial minutes after slurry application, which is explained by its high partitioning into the air phase. Long-term odor effects are therefore assessed to be mainly due to other volatile compounds with low odor threshold values, such as 4-methylphenol. PTR-MS signal assignment was verified by comparison to a photo-acoustic analyzer (NH_3_) and to thermal desorption GC/MS (VOC). Due to initial rapid changes in odorant emissions and low concentrations of odorants, PTR-MS is assessed to be a very useful method for assessing odor following field application of slurry. The effects of treatments on odorant emissions are discussed.

## 1. Introduction

Livestock manure slurry is widely used as organic fertilizer in order to utilize the nutrient value of this waste product from animal production [1]. However, land spreading of livestock slurry is also associated with gaseous emissions. Agriculture is well recognized in Europe to contribute with more than 90% of the total nitrogen emissions, with animal manure constituting the main source of NH_3_ [2,3]. In addition to NH_3_, application of manure is a source of odor nuisance [4] and techniques for reducing odorant emissions, such as direct slurry injection into soil or slurry treatment [4–7] have been introduced. In order to identify suitable odor abatement techniques, reliable odor quantification is necessary. The widely used method of dilution-to-threshold olfactometry suffers from (1) poor recovery of certain odorants in sampling bags [8,9]; (2) poor recovery of certain odorants in the dilution systems of olfactometers [10]; and (3) limited reproducibility and repeatability even if a common standard is used [11–13]. Chemical analysis of odorants is a potential alternative and is important to assess the performance of abatement technologies. In order to develop odorant analysis for legislation and assessment of abatement technologies, more knowledge is needed on odorant composition from different sources and under different conditions. Feilberg *et al.* [5] used a static chamber to investigate emissions of odorants and found several potential key odorants, but also identified shortcomings of the static method. Parker *et al.* [14] recently used a small wind tunnel to investigate emissions of odorous VOC from application of pig slurry. However, their measurement technique had limited time resolution and did not include two potential key odor compounds, H_2_S and methanethiol.

Due to the variation in volatility (e.g., H_2_S *vs.* 4-methylphenol) and chemical stability it is likely, that the odorant/VOC composition of the emissions may vary over time, which is also indicated by the results obtained by Feilberg *et al.* [5]. This may affect which key odorants are expected to cause nuisance at different periods after application and may also affect the choice of relevant abatement technologies.

As an alternative to logistically demanding full scale field trials, emissions can be investigated at laboratory scale using small model systems with an artificial air exchange [15,16], which can be designed to simulate ambient conditions as far as possible [17]. This is useful for investigating emission processes under controlled conditions and for obtaining data on odorant composition for different slurry treatments and application techniques. A range of different manure treatment techniques have been proposed [18–23], and ozonation has been found to reduce emissions from slurry storage to variable degree [19–22]. However, little is known about the effects of treatment on odorant composition and temporal variation in emissions from slurry applied to soil.

The aim of the present study was to provide new time-resolved data on emissions from manure slurry applied to soil with respect to the potential for measurement of key odorants and ammonia by online proton-transfer-reaction mass spectrometry (PTR-MS). Using pig slurry as a case, treatments by ozonation, and solid-liquid separation were applied in addition to untreated slurry. The treatments were included in order to assess if any major changes in chemical composition due to the treatments could be identified.

## 2. Experimental Section

### 2.1. Overall Setup

**Figure 1.**
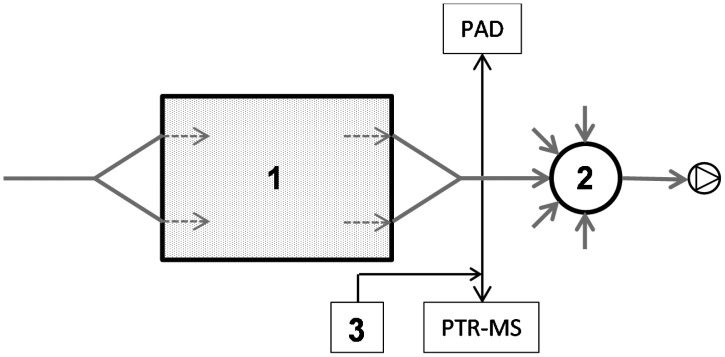
Simplified diagram of the setup used (top-down view). **1**: Dynamic chamber packed with soil; **2**: Air distribution manifold [15] with up to 15 inlets; and **3**: Dilution air for PTR-MS.

The setup used in the present work was identical to the setup used by Nyord *et al.* [15]. A simplified diagram is presented in Figure 1. In short, soil was placed in dynamic chambers, which were rectangular PVC boxes (350 mm long × 250 mm wide × 170 mm high). The boxes were filled with 12 kg of soil to about 65% of their height, leaving a headspace of about 5.2 L. Soil was compacted as described in detail by Dorno *et al.* [16]. Air entered the chamber via two orifices (10 mm in diameter) placed 100 mm apart 50 mm from the top of the chamber. The air had an average temperature of 17.5 °C and was sucked through the dynamic chambers at a flow of 13.6 L·min^−1^ per chamber equal to an air exchange rate of 2.61 min^−1^. The dimensions of the dynamic chamber and the air flow used were close to the setup used by Reichman *et al.* [17] and the air exchange rate was similar to the one used by Parker *et al.* [14], who used 2.36 min^−1^ in a small wind tunnel used as dynamic flux chamber. A preliminary smoke test indicated turbulent conditions in the chamber headspace, implying that the local air velocities were significantly higher than the nominal average air velocity calculated from the air flow and headspace dimensions (0.035 m·s^−1^).

Eight dynamic chambers were run in parallel with duplicates of each treatment including control. Measurements of NH_3_ by photo-acoustic IR (PAD) were done in duplicate and measurements of VOC, H_2_S and NH_3_ by PTR-MS only on one chamber for each treatment. This was done in order to achieve a high time resolution, especially for the initial emissions measured by PTR-MS. Both instruments measured alternately between the eight and four chambers, respectively. The average deviation between duplicate PAD measurements was 13% based on four sets of duplicate chambers (n_total_ = 171). In total, 90% of the deviations in NH_3_ concentration between duplicate chambers were <22%. Graphs showing comparisons of the duplicate PAD measurements are presented in Supplementary Information (Figure S12). Triplicate chambers were used in a preliminary test using acid impingers for collection and subsequent analysis of ammonia concentrations. This gave repeatability standard deviations in the range of 5%–20%.

### 2.2. Soil Preparation

A loamy sand soil (Typic Hapludult) was collected at the experimental field station of Research Centre Foulum (56.5 N, 9.58 E) in late winter 2010, before the sowing of a spring cereal crop. A cubic meter of soil was gathered from 0 to 300 mm depth. One week before each experiment was initiated the soil was passed through a 5 mm sieve to remove roots and stones and subsequently packed in the box to a well-defined bulk density of 1400 kg·m^−3^ by shaking the chamber and pressing the soil as described in details in Dorno *et al.* [16] to a density of 1.4 g·cm^−3^ with a moisture content of 23.3% (vol/vol) at the start of the experiment and 20.1% (vol/vol) at the end. The soil temperature was in average 15.3 °C during the measuring period. Soil pH was measured to be 6.5.

### 2.3. Slurry Treatments and Characteristics

Pig manure from an experimental full scale pig house (Research Center Foulum, Aarhus University) was used for the experiments. The pig manure had a dry matter content of 2%, a content of total ammoniacal nitrogen (TAN; NH_3_ + NH_4_^+^) of 2.8 g/L and an initial pH of ∼8. One part of the slurry batch was separated in a solid and a liquid fraction, which reduced dry matter content to 0.7% in the liquid fraction. The separation was performed on an experimental belt separator, separating 15–40 L of manure per hour, by sequentially adding coagulant (iron(III) sulfate, PIX-113 Kemira Water, Aarhus, Denmark), adding flocculant (C2260 polymer 350 mL 0.25% per liter manure, Kemira Water) and carrying out drainage (200-μm filter) on a filter belt. This has been tested to give the best combination of large floc size and fast dewatering for the flocculated sample as well as the lowest turbidity and smallest dry matter content of the liquid fraction [24]. One part of the liquid fraction of the flocculated slurry and one part of the untreated manure were each treated by ozonation at a dose of 150 mg O_3_ per liter slurry. At this dose, significant reduction in emissions has been observed [20]. Ozone was generated by passing an oxygen stream through an electrical discharge ozone generator (Degrémont Technologies—Triogen, Ozonia LAB2B, Glasgow, UK), set to highest ozone output level (∼45 mg O_3_/L O_2_). The ozone-enriched oxygen stream was added through a stainless steel diffuser placed 20 cm below the manure surface. Ozone was added by a gas flow rate of 200 mL/min during constant stirring, to a manure volume of 1.5 L in a 2 L graduated cylinder.

To summarize, the three treated slurries were: the liquid fraction of separated slurry (SEP), ozonated slurry (OZON) and the liquid fraction of separated slurry treated by ozonation (SEP-OZON). One batch of control slurry (untreated manure; RAW) and the three treated batches of slurry were prepared one day before addition to the dynamic chambers. Prior to addition of slurry to emission chambers, pH was measured to be as follows, RAW: 8.22, OZON: 8.27, SEP: 7.63, SEP-OZON: 8.01.

Slurry (0.2 L) was added manually to each of the dynamic chambers equal to 6.4 g·N·m^−2^. The chambers were immediately closed and sealed after slurry was applied to the soil surface.

### 2.4. PTR-MS Measurements

Measurement of odorous gases was carried out by a high sensitivity PTR-MS (Ionicon Analytik, Innsbruck, Austria), which is an online technique using protonated water to ionize compounds with a proton affinity higher than water [25,26]. Selected m/z values (Table 1) were monitored over 20 h by PTR-MS in multiple ion detection mode (as opposed to full mass scan mode) in order to achieve the lowest detection limits and the highest time resolution. The ions were selected based on previous work on very similar systems [5,21,27,28]. In addition, a series of mass scans (from m/z 21 to m/z 140) were performed 75–80 min after slurry application using a dwell time of 0.5 s per m/z.

The sampling for the PTR-MS was done by a thermostatic valve system (50 °C) consisting of 5 on/off valves controlled by the PTR-MS instrument. All sampling tubes were insulated and heated to 50 °C. In order to avoid too high NH_3_ and humidity levels in the analyzed air, the sampling air from the chambers were diluted (1:1) by charcoal-filtered (Supelco, Bellefonte, PA, USA) room air.

Initially, the measurements were cycled between the four different treatments every 2 min and, hence, each chamber was measured every 8 min. The measurement dwell time was 0.5 s for each m/z value except for m/z 18, which was set at 0.1 s due its high abundance. This gave 9 or 10 measurements on each channel of which the first 4 as well as the last one were discarded in order to ensure a stabilized signal. Prior to addition of slurry to the chambers, the room air was analyzed by PTR-MS in order to correct data for any contribution from the background air sucked into the chambers. The background measurement was done again 75 min after the slurry application. After this initial period, the measurement program was changed to measuring 5 min on each of the four chambers. In addition, room air was added to the measurement cycle in order to continuously correct for background contributions. Thus, every chamber was measured at 25 min intervals from ∼80 min after slurry addition until the end of the experiment (20 h).

The PTR-MS was run under standard drift tube conditions (2.15 mbar, 333 K) using a drift voltage of 600 V, which gives an E/N number (electric field per gas density [31]) of 138 Townsend. The PTR-MS was calibrated with respect to sulfur compounds (H_2_S, dimethyl sulfide and methanethiol) by means of certified reference gases at known concentrations (±10%) close to 5 ppm. The reference gases were diluted with clean air by using calibrated mass flow controllers (Sierra Instruments, Monterey, CA, USA). Humidity dependent H_2_S calibration is necessary and was performed as described by Feilberg *et al.* [27]. For the remaining compounds, the sensitivity was estimated based on calculated or experimentally determined proton-transfer rate constants [32,33]. Calculated rate constants were obtained as recommended by Cappellin *et al.* [34], who demonstrated accuracy within ±10% with this approach. If relevant, concentrations were corrected for fragmentation based on known fragmentation patterns [28]. A mass transmission curve was measured before and after the experiment as described previously [27].

A relatively high background signal at m/z 18 is present, which adversely affects the detection limit of PTR-MS towards NH_3_ (see Table 1) due to a relatively high instrumental background signal. For this study, this was not a problem since the NH_3_ concentrations were observed to be in the range of 0.9–25 ppm. For NH_3_, a proton transfer rate constant of 2.15 × 10^9^ cm^3^·s^−1^ was calculated by the method recommended by Capellin *et al.* [33], which agrees well with the measured value of 2.2 × 10^9^ cm^3^·s^−1^ reported by Lindinger *et al.* [31]. When the highest levels of ammonia of ∼10 ppm (after dilution) occurred, this resulted in a 5%–10% lower abundance of the primary ion as compared to low levels of ammonia. In addition, the electron multiplier response levels off at high ion abundance due to saturation of the detector. In the present study, maximum NH_3_ raw signals of ∼1.5 × 10^6^ counts per second were observed at the highest level (shortly after slurry addition).

### 2.5. Photo-Acoustic Measurements

NH_3_ was additionally measured by an Innova Photo-acoustic Field Gas monitor 1312 (PAD) coupled with a multipoint sampler 1303 (both Lumasense Technologies, Ballerup, Denmark). Via a 3.5 m long polytetrafluoroethylene (PTFE) tube (internal diameter 3 mm), part of the air flow was pumped from the chamber outlet to the PAD, where the NH_3_ concentration was determined every 65 s. The PAD monitored the NH_3_ concentration in the air five successive times on the same chamber before changing channel to the next box. Only the last measurement was used due to time delay in the PAD response [35].

### 2.6. GC/MS Measurements

Supplementary screening of VOC in the headspace above identically treated or untreated batches of manure was carried in a separate experiment. The analyses of these were done by gas chromatography with mass spectrometric detection (GC/MS) on samples collected by adsorption tubes packed with a Tenax TA/Carbograph 5TD combination as described in [36]. Samples were collected at 100 mL·min^−1^ for 10 min. Concentrations of selected compounds were estimated based on 1-point calibrations by adding aliquots of compound solutions (in pentane or methanol) to the sorbent tubes and flushing the solvents by a flow of helium. It should be noted that the headspace samples for TD-GC/MS were collected shortly after treatment, whereas the soil application experiments were done one day after the treatments.

## 3. Results and Discussion

A dataset consisting of gas concentrations and emissions as a function of time was obtained for each of the batches tested. Emissions from each batch were determined from measured concentrations and air exchange rate. In the following discussion, the results (including effects of treatment) are grouped according to the following compound groups: (1) Nitrogen compounds; (2) sulfur compounds; (3) carboxylic acids; (4) alcohols; (5) carbonyl compounds (aldehydes and ketones) and (6) aromatic compounds (phenols and indoles). Ranking of the odorants with respect to overall odor contribution is treated in a separate section. It is beyond the scope of this paper to make a general assessment of the effects the treatments, which would require more different slurry batches to be tested. However, preliminary information on the effects of treatments are discussed for each group of compounds.

Assignment of m/z values to specific compounds were based on existing knowledge on emissions from slurry and livestock production [5,14,21,27,28,37] combined with TD-GC/MS measurements carried out on the headspace of treated and untreated slurry in a separate experiment using the same raw manure and the same treatments. The TD-GC/MS results are presented in Section 3.7 and Supplementary Information (Table S3 and Figures S8, S9, S10 and S11).

### 3.1. Emissions of Ammonia and Trimethylamine

**Figure 2.**
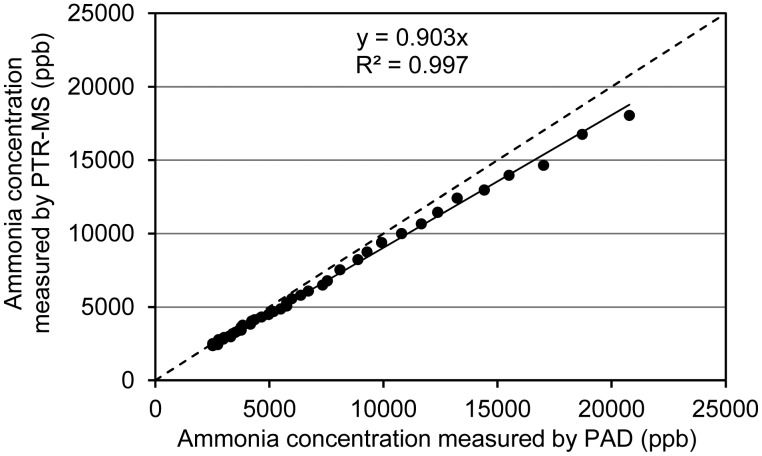
Comparison of ammonia measurements by PTR-MS and PAD. The solid line is a linear regression curve.

NH_3_ was by far the most abundant gaseous compound measured in the emissions from the slurry applications. NH_3_ was measured by both PAD and PTR-MS. PAD and PTR-MS concentration measurements are compared in Figure 2. As can be seen, a good correlation between the two methods is observed although the PTR-MS results on average are ∼10% lower than the PAD measurements. As mentioned in the previous section, detector saturation and reduced primary ion levels possibly affects the PTR-MS measurements in the concentration range above ∼5 ppm, which may partly explain the deviation. It should be noted, however, that the deviation is within the uncertainty of the calibration, which is determined by the uncertainty of the rate constant used, the uncertainty of the reference gas concentration used for measuring transmission (10%) and uncertainty in the dilution factor (assessed to be ∼5%). Below ∼5 ppm PTR-MS and PAD data agrees very well and the estimates of total emissions based on PTR-MS and PAD over the 20 h measurement period are strikingly close (Supplementary Information; Table S1).

**Figure 3.**
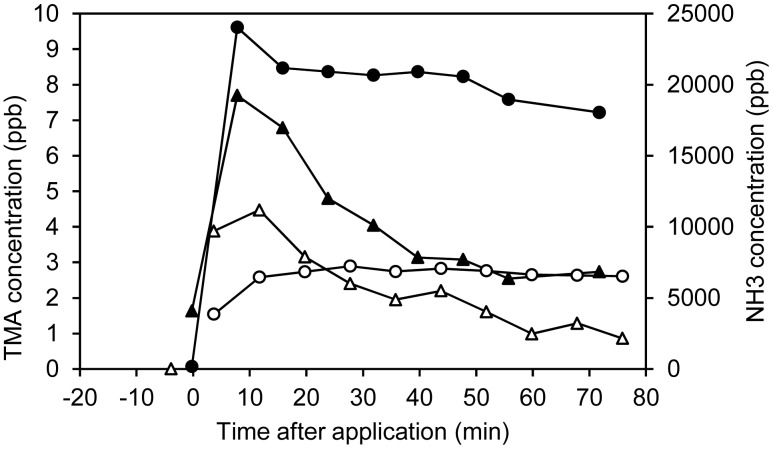
Comparison of RAW (▲) and SEP (△) trimethylamine concentrations with RAW (●) and SEP (○) NH_3_ concentrations in the initial 75 min after application of slurry to the soil.

In addition to ammonia, a signal at *m/z* 60 assigned to trimethylamine was observed. This signal was corrected for contribution of the ^13^C isotopomer of acetone (*m/z* 59). This correction was <10% of the *m/z* 60 signal for raw manure. The maximum trimethylamine concentration observed was ∼9 ppb. Trimethylamine was not detected by TD-GC/MS, but this was expected since it has previously been observed in that trimethylamine only elutes from the specific GC column in the first few runs of a column (not published). Examples of temporal trends in NH_3_ and trimethylamine emissions are presented in Figure 3. As can be seen, the emissions of trimethylamine are in the order of 1000–3000 times lower than the ammonia emissions. Furthermore, the trimethylamine concentration decreases at a faster rate than the ammonia concentration which is relatively stable in the first 75 min. The equilibrium air-water distribution of trimethylamine and ammonia are comparable when taking into account both the Henry's law constants of trimethylamine and ammonia (9.6 M/atm and 58 M/atm [38]) and the pKa values of trimethylammonium and ammonium (9.8 and 9.25). Thus, the faster decrease (in comparison to ammonia) cannot be explained by a faster physical stripping of the compound, but must be due to other causes, e.g., biological oxidation. For both NH_3_ and trimethylamine, lower emissions were observed for SEP and SEP-OZON, which can be explained by lower pH in the liquid fraction after separation. In addition, the lower content of dry matter is expected to increase infiltration [39], which will also reduce emissions. Ozonation of the liquid fraction after separation resulted in lower emissions of trimethylamine, whereas NH_3_ was not affected by ozonation. This can be explained by a much faster reaction of ozone with trimethylamine compared to NH_3_ [40,41] and the reason for not seeing this trend in non-separated slurry most likely is due to a higher consumption of ozone by other reactants in raw slurry, including dry matter.

### 3.2. Emissions of Sulfur Compounds

**Figure 4.**
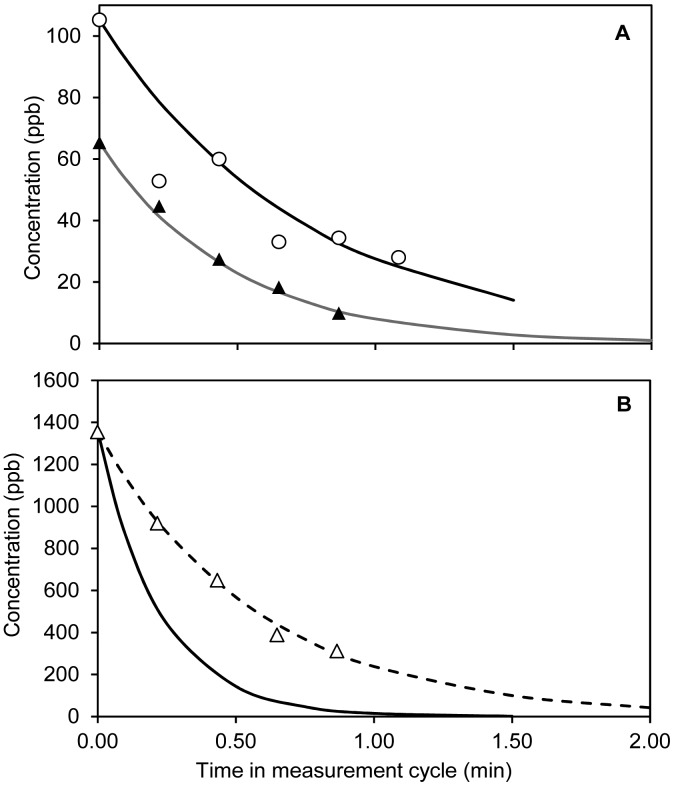
(**A**) Decay curves of H_2_S for RAW (○) and SEP-OZON (▲). Solid lines are maximum stripping rates (Equation (1)) based on measured pH and air flow; (**B**) Decay curve of H_2_S for SEP (△). The solid line is the maximum stripping rate (Equation (1)) and the dashed line is the maximum stripping rate based on adjusting pH of SEP from 7.63 to 8.1.

Sulfur compounds were emitted in relatively low quantities from the manure distributed in the soil-packed dynamic chambers and were only detected in the initial ∼1 h of the experiment. H_2_S was initially detected at high concentrations, but disappeared so fast that a proper concentration-versus-time profile could not be constructed and the total emissions could not be quantified. In addition to H_2_S, methanethiol and dimethyl sulfide could also be detected at concentrations not exceeding 2 ppb (see Supplementary Information, Figure S3). Due to the fast detection by PTR-MS, it is possible to present H_2_S measurements within the first measurement period in three out of four chambers. Within each 2 min measurement cycle, the concentrations declined rapidly and averaging of the data is therefore not relevant. This rapid decay of H_2_S concentrations in the initial phase after slurry application as can be seen from Figure 4. A clear decay was not seen in the ozonated raw slurry, since H_2_S emission in this case was already low (∼20 ppb) at the first measurement point ∼5 min after slurry application.

The fast decays of H_2_S emissions can be ascribed to both physical stripping (by air) due to the relatively low water solubility of H_2_S and oxidation of H_2_S upon contact with air after application to the soil surface. In Figure 4, the maximum decay rates due to physical stripping have been included. The maximum stripping rates have been estimated assuming instant equilibrium, *i.e.*, no mass transfer limitation, by the following equation:

(1) $$C(t)=C(0)\cdot e^{\left(-Q\cdot \alpha \cdot \frac{K_{H}}{\text{RTV}}\cdot t\right)}$$

The term α is used to account for the pH dependent dissociation of H_2_S in the liquid phase:

(2) $$\alpha =\frac{1}{1+10^{\left(pH-\text{pKa}\right)}}$$

*C*(*t*) is concentration in ppb, *C*(0) is the initial concentration in the measurement cycle, *Q* is the air flow through the flux chamber in L/min, *V* is the volume of applied liquid, *K_H_* is the Henry's law constant of neutral H_2_S in atm L·mol^−1^, R is the gas constant (0.08206 L atm mol^−1^·K^−1^) and T is temperature in K. For these calculations, the initial bulk pH was used and no correction for any effect of ionic strength was included as this was unknown.

In two out of the three cases (RAW and SEP-OZON) the calculated decays are strikingly close to the observed decays. In the third case, SEP, the observed decay is slower than the calculated decay. A possible explanation for SEP is that pH, which initially was lower than the others (7.63), increased during slurry application due to loss of CO_2_ at the surface. Adjusting pH of the SEP batch to 8.1 brings the theoretical and experimental data in close agreement (Figure 4).

It should be mentioned that microbial or chemical surface oxidation of H_2_S may also affect the decay. Microbial oxidation of H_2_S at the manure-air surface has been observed to occur at turnover rates of a few seconds [42,43], which is significantly faster than typical chemical oxidation [43]. However, it would be expected that some time would be necessary for such a surface microbial H_2_S oxidation to become active. Due to the rapid decay of H_2_S, it is not possible to assess the effects of treatment on H_2_S.

### 3.3. Emissions of Carboxylic Acids

**Figure 5.**
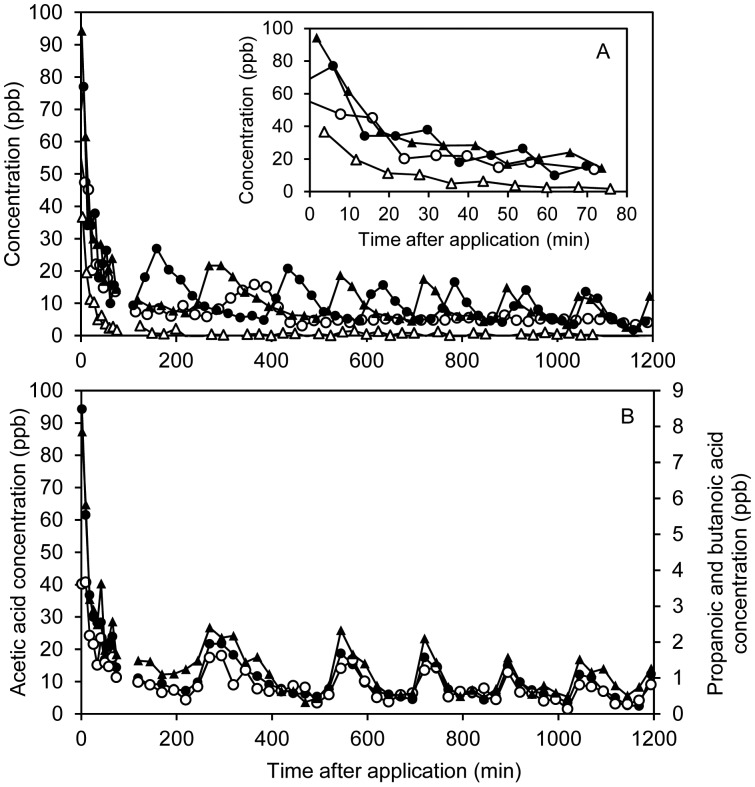
(**A**) Acetic acid concentrations as a function of time for RAW (○), SEP (△), OZON (●) and SEP-OZON (▲). Insert: Initial 75 min; (**B**) Comparison of temporal patterns of concentrations of acetic acid (●), propanoic acid (▲) and butanoic acid (○) for OZON-SEP.

C_2_–C_5_ carboxylic acids were detected and monitored by their protonated molecular ions (MH^+^) corrected for fragmentation by water elimination corresponding to loss of *m/z* 18 (see Table 1). Based on the TD-GC/MS headspace measurements, the carboxylic acids consist mainly of linear-chain compounds except for C_5_, which appear to consist mainly of 3-methylbutanoic acid. Normally, pentanoic acid is expected to dominate over 3-methylbutanoic acid [27], but it should be noted that the levels were very low and close to the detection limit. All carboxylic acid concentrations decayed by a factor of 3–5 in the first 75 min period. One example, acetic acid is shown in Figure 5A and further examples are seen in Supplementary Information (Figures S4,S5). Due to the low air-water partitioning coefficient, high pH and aqueous dissociation of the acids, a reduction in the slurry concentration by evaporation/stripping can be neglected over this time scale. The reduced emissions over time are therefore ascribed to a combination of microbial oxidation of acids upon contact with air and slurry infiltration, which transports compounds away from the surface and thus reduce the emission potential.

A surprising temporal pattern in emissions of acids can be seen in Figure 5A,B. Following the initial decay in concentration, several incidences of bursts of emissions lasting 1–2 h are seen. These bursts are only seen for the acids and coincide for different acids in the same batch (Figure 5B), but not for the different treatments (Figure 5A and Supplementary Information, Figures S4,S5). Thus, it cannot be ascribed to temperature fluctuations or other external fluctuations, which would have been the same for all chambers. Fluctuations in the air flow would have affected all compounds. Based on these observations, the most likely explanation for the patterns is that the dissolved carboxylic acids encounter different pH levels in the heterogeneous soil microenvironments as the slurry is transported down through the soil. Soil microenvironments with low pH will lead to a strong increase in the partitioning of carboxylic acids into the gas phase.

In addition to C_2_-C_5_ acids, a signal at m/z 47 was observed that may be assigned to formic acid or ethanol. Formic acid may be produced from ozonation [44] and was observed in the TD-GC/MS headspace pre-test at higher levels than ethanol, which was, however, also observed. Based on the current data it is not possible to conclude whether m/z 47 is mainly due to formic acid or ethanol, but both compounds are present.

Apparently, separation of slurry leads to strongly reduced emissions of carboxylic acids (Figure 5 and Table S1). Carboxylic acids are highly water soluble and are expected to be retained in the liquid fraction from solid-liquid separation. Hence, faster infiltration of the liquid fraction is more likely to be an influencing factor rather than the separation process, but this issue requires further investigations. On the other hand, ozonation leads to increased emissions of acids, especially in the case of SEP *vs.* SEP-OZON, which is expected, since ozonation of wastewater has been shown to produce carboxylic acids [44].

### 3.4. Emissions of Aromatic Compounds (Phenols and Indoles)

**Figure 6.**
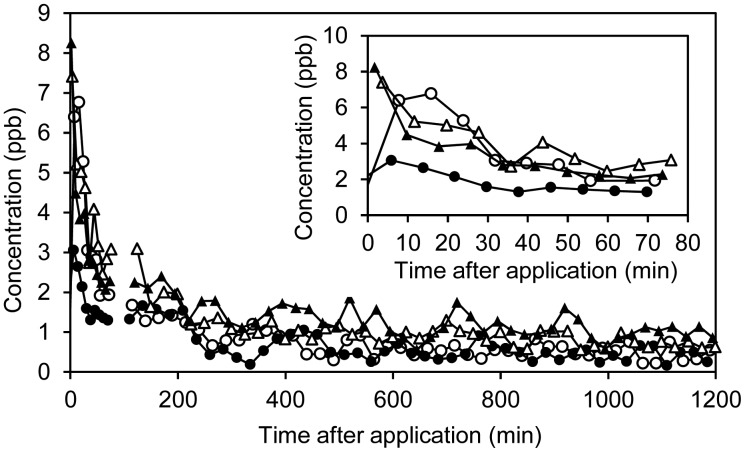
Concentrations of 4-methylphenol as a function of time for RAW (○), SEP (△), OZON (●) and SEP-OZON (▲).

Masses corresponding to phenol (*m/z* 95), 4-methylphenol (*m/z* 109), 4-ethylphenol (*m/z* 123), indole (*m/z* 118) and 3-methyl-1*H*-indole (*m/z* 132) were monitored by PTR-MS throughout the experimental period. Indole and 3-methyl-1H-indole were only measured to be present at very low levels (less than 0.4 ppb). These five aromatic compounds were identified by TD-GC/MS as well and other compounds that could contribute to these masses were not detected. Dimethyl disulfide could potentially contribute to *m/z* 95 and has been reported previously from livestock manure based on GC/MS measurements [45,46], but as discussed in the literature [47–50], alkyl thiols are easily converted to disulfides (and even trisulfides) by oxidative dimerization during sampling, sample storage and analysis. Dimethyl disulfide gives rise to a fragment at *m/z* 79 (CH_3_S_2_^+^) in PTR-MS analysis [51], but this mass was not detected in the scan mode measurements (80 min after slurry application). It should be noted, though, that *m/z* 95 was also low at this stage. Dimethyl disulfide was detected by TD-GC/MS, but this is ascribed to dimerization of methanethiol, which takes place under the analytical conditions applied [49]. In conclusion, we assign *m/z* 95 primarily to phenol. This is in line with Feilberg *et al.* [27], who concluded that the PTR-MS signal at *m/z* 95 in a livestock house emission primarily was due to phenol rather than dimethyl disulfide. Selected results for 4-methylphenol are presented in Figure 6. It is seen that the concentrations initially decrease relatively rapidly, but the compound is detected throughout the measurement period.

Small (∼20%) emission reductions by ozonation of raw manure were seen for 4-methylphenol and 4-ethylphenol (Table S1). However, the emission in the initial phase was significantly reduced to a larger degree (by 50%) in the OZON batch (Figure 6, insert). The rate constants for the reactions of ozone with phenol and 4-methylphenol are 1.3 × 10^3^ M^−1^·s^−1^ and 3 × 10^4^ M^−1^·s^−1^, respectively [52] and the rate constants of the corresponding phenolate ions occur at close to diffusion controlled rate, e.g., 1.4 × 10^9^ M^−1^·s^−1^ for unsubstituted phenolate [52]. Since the pKa values of phenol and 4-methylphenol are 9.9 and 10.3, respectively, the reactions of the phenolate ions should dominate over the reactions of non-dissociated phenols at the pH values of the present study. In previous studies [19] that demonstrated degradation of 4-methylphenol by ozonation of stored slurry, higher doses of ozone were used.

On the other hand, emissions of phenols from the liquid fraction of separated slurry were ∼2 times higher than from non-separated slurry (Table S1). The reason for this is not clear, but it can be speculated that the mechanical separation of the manure leads to a transfer of phenols from particulate matter (adsorption) to the aqueous phase, which would increase the emission potential.

### 3.5. Emissions of Alcohols

Measurement of alcohols by PTR-MS is complicated by fragmentation due to dehydration of the initially formed protonated molecule [53]. This does not occur for methanol, but for ethanol and higher alcohols it needs to be taken into account. For ethanol the protonated parent molecule (m/z 47) is still observed, but for higher alcohols this is not the case [53]. e.g., for 1-propanol, only m/z 43, 41 and 39 are observed at standard E/N-values:

$$\begin{array}{c} {\text{CH}}_{3}{\text{CH}}_{2}{\text{CH}}_{2}\text{OH}+{\text{H}}_{3}{\text{O}}^{+}\to {\text{CH}}_{3}{\text{CH}}_{2}{\text{CH}}_{2}\text{O}{{\text{H}}_{2}}^{+}(\text{m}/\text{z}\;61)\to {\text{CH}}_{3}{\text{CH}}_{2}\text{C}{{\text{H}}_{2}}^{+}(\text{m}/\text{z}\;43)+{\text{H}}_{2}\text{O}\\ {\text{CH}}_{3}{\text{CH}}_{2}\text{C}{{\text{H}}_{2}}^{+}\to {\text{C}}_{3}{{\text{H}}_{5}}^{+}(\text{m}/\text{z}\;41)+{\text{H}}_{2}\to {\text{C}}_{3}{{\text{H}}_{3}}^{+}(\text{m}/\text{z}\;39)+2{\text{H}}_{2} \end{array}$$

Detection of alcohols other than methanol/ethanol must therefore be done with great care as a number of interferences are possible. In this experiment, *m/z* 47 (ethanol and formic acid as discussed in the previous section) were abundant in all batches. In addition, *m/z* 39, 41, 43 and 57 were detected by full scan measurements ca. 80 min after slurry application in all batches. Of these, *m/z* 43 is mainly ascribed to acetic acid fragmentation since *m/z* 61 and *m/z* 43 are highly correlated (*R*^2^ = 0.89, data not shown) with a ratio within the expected range [27]. The other masses may occur due to a number of alcohols; 1- and 2-propanol, 1- and 2-butanol, 2-methyl-1-propanol and 2-methyl-2-propanol. Of these, 1- and 2-propanol and 1-butanol have previously been identified by GC/MS in pig production facilities [27,54]. Relatively high levels of *m/z* 57 were observed (See Table S1; Supplementary Information). M/z 57 was monitored continuously and is tentatively assigned to dehydration of 1-butanol [53]. In addition, propanoic acid fragmentation contributes to *m/z* 57 and the results have been corrected for this based on the fragmentation pattern at the E/N value used. A graph of *m/z* 57 *vs.* time is presented in Supplementary Information (Figure S6).

**Figure 7.**
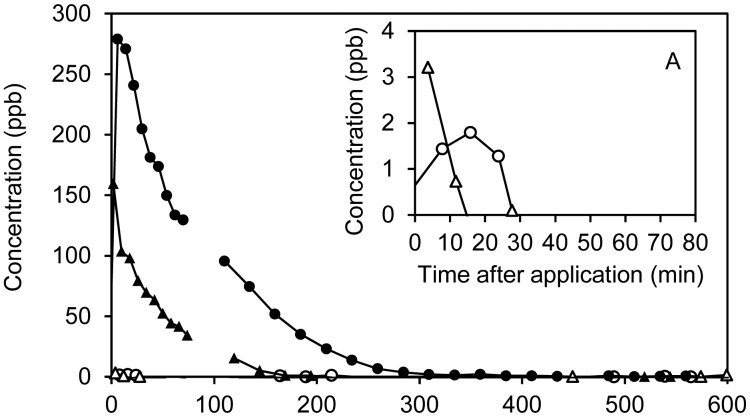

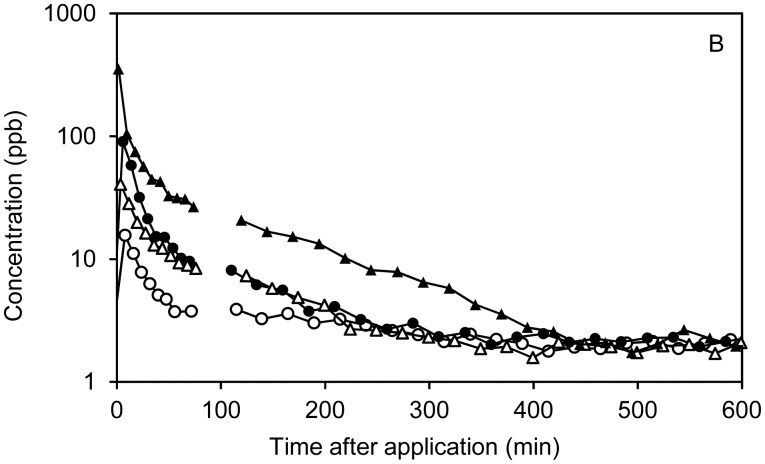
(**A**) Methanol concentrations as a function of time; (**B**) Acetone concentrations as a function of time. Only the initial 600 minutes are presented for RAW (○), SEP (△), OZON (●) and SEP-OZON (▲).

Methanol was only detectable in the raw manure and separated slurry batches within the initial 30 min at low levels of <2 ppb (Figure 7A). However, in both ozonated slurry batches levels of >100 ppb were observed in the emissions (Figure 7A). In total, emissions of methanol were highest for ozonated raw manure followed by the liquid fraction of separated manure (See Table S1; Supplementary Information). The results clearly demonstrate that methanol is a byproduct of ozonation. The fact that highest methanol emission is observed for raw manure indicates that methanol to some extent can be produced from ozone reactions with organic particulate matter in the slurry.

### 3.6. Emissions of Carbonyl Compounds (Ketones and Aldehydes)

Relatively high levels of *m/z* 45 and *m/z* 59 were observed in emissions from all chambers. Based on TD-GC/MS data, *m/z* 45 is assigned to acetaldehyde and *m/z* 59 is assigned to acetone. The emissions declined within the initial 75 min and acetaldehyde was not detectable after ∼50 min (Supplementary Information, Figure S7) whereas acetone emissions remained albeit at low levels during the experiment (Figure 7B). For both raw manure and the liquid fraction from separation, ozonation led to an increase in acetone emissions, indicating that acetone is a byproduct of ozonation. Propanal, if present, would also contribute to *m/z* 59, but this compound was not found in any of the GC/MS screenings. Acetaldehyde is also indicated to be produced by ozonation, but the rapid decay makes it more difficult to assess the effect accurately (Supplementary Information, Figure S7).

In addition to the acetaldehyde and acetone masses, *m/z* 73 and *m/z* 87 were monitored and these mass signals can be ascribed to several carbonyl compounds. Both masses exhibited elevated levels in the ozonated batches indicating that these are partly ozonation byproducts. Based on TD-GC/MS data, compounds contributing to *m/z* 73 are mainly butanone and butanal, with the former appearing in higher concentrations. Compounds assigned to *m/z* 87 (from GC/MS data) are mainly C_5_-aldehydes (pentanal, 2- and 3-methylbutanal) and 2,3-butanedione. For both PTR-MS and GC/MS data, aldehydes are consistently present at higher levels in ozonated compared to non-ozonated samples reflecting that these compounds are byproducts of ozonation. From the TD-GC/MS data (Supplementary Information, Figure S10) it is seen that aldehydes containing up to 10 carbon atoms were detected in samples from ozonated batches. In the literature, aldehydes containing up to nine carbon atoms have been reported to be ozonation byproducts [55,56], which is consistent with the data presented in this paper. Masses corresponding to aldehydes containing more than five carbon atoms were not continuously monitored by PTR-MS, but several expected aldehyde masses (*m/z* 69, 83, 97, 101, 111, and 115) were observed in the mass scans obtained after the initial 75 min (Supplementary Information, Table S2), mainly in samples from chambers containing ozonated slurry (separated or non-separated).

### 3.7. TD-GC/MS Data

**Figure 8.**
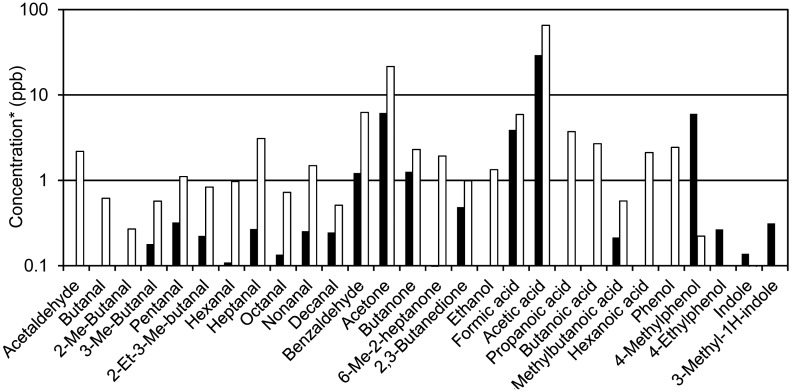
Examples of TD-GC/MS data used for verifying mass assignments of PTR-MS data. **White**: ozonated slurry; **Black**: untreated slurry. *Concentrations were estimated based on either authentic standards or surrogate standards (see Supplementary Information, Table S3).

Selected results from TD-GC/MS measurements are shown in Figure 8. Only compounds that are unambiguously detected above the detection limit as well as background and blank levels are included. Sulfur compounds are not measured by this method. Additional data is included in Supplementary Information (Figure S8, S9, S10 and S11). It should be noted that the samples were collected prior to the slurry application experiments in headspace above the slurry samples. The data was used mainly for the assignment of m/z values observed in PTR-MS to specific compounds.

### 3.8. Contribution to Odour

**Figure 9.**
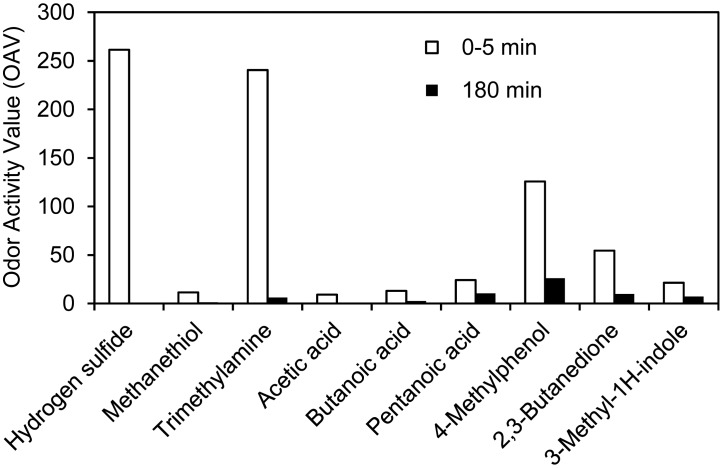
Compound specific odor activity values estimated immediately after slurry (RAW) application and after 180 min.

Based on the concentrations (C) of odorants and literature data on odor threshold values (OTV), an estimate of the contribution of the different odor compounds can be made. The odor contributions are expressed as odor active values (OAV): OAV = C/OTV. In Figure 9, OAVs are presented for compounds with OAV > 1 shortly after application of untreated slurry and after 180 min. The comprehensive OTV dataset by Nagata [29] has been used for this purpose. This dataset was selected based on the following: (1) It is to our knowledge the largest existing OTV dataset (obtained by the same method) consisting of 223 compounds; (2) it is based on a large number of individuals; (3) all OTVs are determined by the same method; and (4) Quality assurance is well documented. Most notably, the specific exposure concentrations were measured by gas chromatography as a part of the quality assurance. Some of the OTVs are relatively low compared to older values [30], especially for less volatile and/or polar compounds such as acids, amines and phenols. This is most likely a consequence of the improved quality assurance in terms of measured exposure concentrations. For less polar volatile compounds (e.g., H_2_S and 1-butanol), the OTVs agree well with earlier values.

It can be seen that the initial odor is estimated to be dominated by H_2_S and trimethylamine. Since these decay relatively fast, however, the odor contribution after 180 min is shifted towards a higher (relative) contribution by mainly 4-methylphenol. The initial contribution of H_2_S is difficult to assess, since the emissions cease rapidly. Transient concentrations up to ∼1000 ppb were measured and therefore odor may initially be completely dominated by a pulse of H_2_S. The results can be compared with recent data from a pig production facility, where odor was predicted by a semi-field statistical method to be caused mainly by H_2_S, methanethiol, 4-methylphenol and trimethylamine [57]. In our work, methanethiol concentration was very low, but otherwise the results are strikingly similar in terms of key odorants. 4-methylphenol has also been suggested by other researchers as a key odorant from pig production [46]. It should be noted that the contribution of 2,3-butanedione is somewhat uncertain due to the possible contribution of other compounds to *m/z* 87 (*i.e.*, aldehydes) with slightly higher OTV [29].

The observed increased emissions of carboxylic acids and aldehydes by ozonation may lead to increased odor, but due to the complexity of the odorant composition, this is not straightforward to evaluate. The results also imply that application of sensory odor analysis (bag sampling combined with dynamic dilution olfactometry) for this type of emission would have very limited use because compounds such as trimethylamine, 4-methylphenol, 3-methyl-1*H*-indole and carboxylic acids are associated with very poor recovery in the sampling bags [8,9] and at least in one type of dilution system [10] used for sensory evaluation. As a consequence, there is a strong bias towards increased contribution of volatile sulfur compounds in olfactometry. These compounds (*i.e.*, H_2_S), however, mainly contribute to odor in the initial few minutes after slurry application.

## 4. Conclusions/Outlook

It is demonstrated that the composition of odorants following application of pig slurry to soil changes over time with a contribution of volatile sulfur compounds (especially H_2_S) in the first minutes after application. 4-Methylphenol is in this case observed to be the major odorant at longer post-application times. PTR-MS is demonstrated to be a very useful tool for this purpose providing the ability to measure all odorants known to be relevant at high time resolution. The laboratory scale setup used in the current work can be used for future systematic studies of the effects of slurry treatment on gaseous emissions.

## Supplementary Material



## Figures and Tables

**Table 1.** Monitored m/z values, compound assignments, detection limits (3 standard deviations of blank values) and odor threshold values (OTV).

**Mass-to-Charge (m/z)**	**Compound^1^**	**DL (ppb)**	**OTV (ppb)^2^**
18	NH_3_	52	1,500
33	Methanol	1.1	33,000
35	H_2_S	5.0	0.7
43	Acetic acid (fragment)	0.8	
45	Acetaldehyde	1.0	1.5
47	Formic acid/ethanol	1.7	4400/520
49	Methanethiol	0.05	0.07
57	1-Butanol (fragment)	0.44	38
59	Acetone	0.31	42,000
60	Trimethylamine ^3^	0.16	0.032
61	Acetic acid	0.83	6.0
63	Dimethyl sulfide	0.19	3.0
71	Butanoic acid (fragment)	0.19	
73	Butanone	0.09	440
75	Propanoic acid	0.18	5.7
87	2,3-Butanedione	0.22	0.05
89	Butanoic acid	0.14	0.19
95	Phenol	0.17	5.6
103	C_5_ carboxylic acids	0.12	0.037/0.078 ^4^
109	4-methylphenol	0.13	0.054
118	Indole	0.08	0.3
123	4-Ethylphenol	0.14	1.6 ^5^
132	3-Methyl-1*H*-indole	0.05	0.0056

1 See text for discussion of compound assignment;

2 Data from [29] except where indicated;

3 Corrected for the ^13^C isotopomer of acetone;

4 Values for 1-pentanoic acid and 3-methyl-butanoic acid;

5 Estimated value [27] based on van Gemert [30].
