# Supplementary material for: Application of PTR-MS for Measuring Odorant Emissions from Soil Application of Manure Slurry
Source: Sensors (Basel). 2015 Jan 9;15(1):1148–67. doi: 10.3390/s150101148 (PMC4327069; doi:10.3390/s150101148)
Supplement: Supplementary file 1 [file sensors-15-01148-s001.pdf]

OPEN ACCESS

sensors

ISSN 1424-8220

www.mdpi.com/journal/sensors

Supplementary Information

Application of PTR-MS for Measuring Odorant Emissions from Soil Application of Manure Slurry. *Sensors* 2015, *15*, 1148-1167

Anders Feilberg †,*, Pernille Bildsoe † and Tavs Nyord †

Aarhus University, Department of Engineering, Hangøvej 2, DK-8200 Aarhus N, Denmark;

E-Mails: pernille.bildsoe@eng.au.dk (P.B.); tavs.nyord@eng.au.dk (T.N.)

† These authors contributed equally to this work.

* Author to whom correspondence should be addressed; E-Mail: af@eng.au.dk;
Tel.: +45-3089-6099.

Table S1. Total emissions as a function of treatment for compounds emitted in highest quantities.

	pH	NH_3 (mg)	Loss of TAN (%)		TMA (µg)	HAc (µg)	HPr (µg)	HBu (µg)	HPe (µg)	MeOH (µg)	m/z 47 (µg)	m/z 57 (µg)	Acetone (µg)	m/z 73 (µg)	m/z 87 (µg)	Phenol (µg)	4MP (µg)	4EP (µg)	VOC (mg)
			PTR-MS	PAD															
Raw slurry (RAW)	8.22	183.6	33	32.8	21.5	680	111	78.0	43.7	1.4	59.7	120	254	85.8	94.2	165	128	61.7	1.9
Ozonated raw slurry (OZON)	8.27	201.8	36	36.7	22.7	995	154	100	56.9	988	69.0	129	405	106	148	164	101	48.8	3.5
Separated slurry (SEP)	7.63	63.2	11	15	12.1	87.4	18.4	46.6	39.8	ND	81.1	324	317	61.6	14.6	293	180	88.2	1.6
Ozonated separated slurry (SEP-OZON)	8.01	71.6	13	14.4	5.2	982	138	104	59.5	271	90.0	379	639	132	124	300	199	100	3.5

Abbreviations: TMA, Trimethylamine; HAc, Acetic acid; HPr, Propanoic acid; HBu, Butanoic acid; MeOH, methanol; 4MP, 4-methylphenol; 4EP, 4-ethylphenol.

1. Additional PTR-MS Data

1.1. Nitrogen Compounds

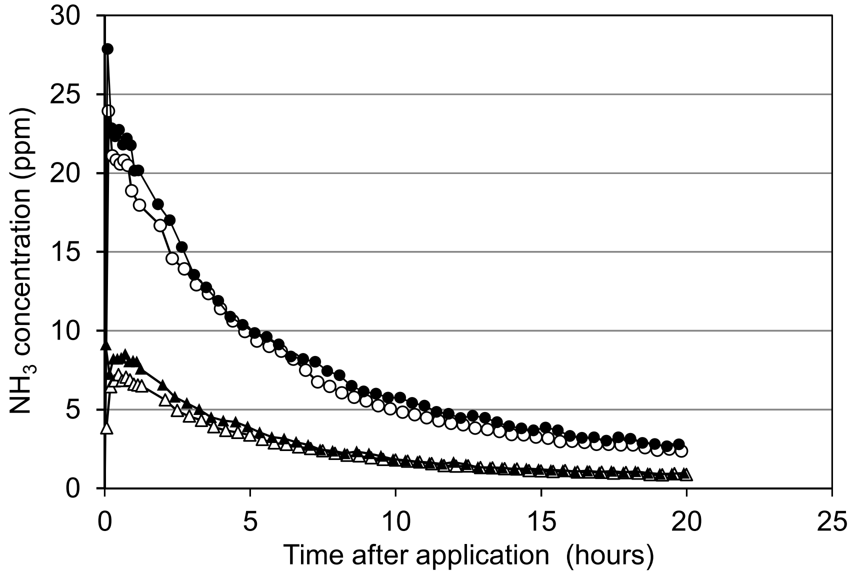

Figure S1. NH_3 concentration profile over 20 h for ozone treated raw slurry (●), raw slurry (○), separated slurry (△) and ozonated separated slurry (▲), measured by PTR-MS.

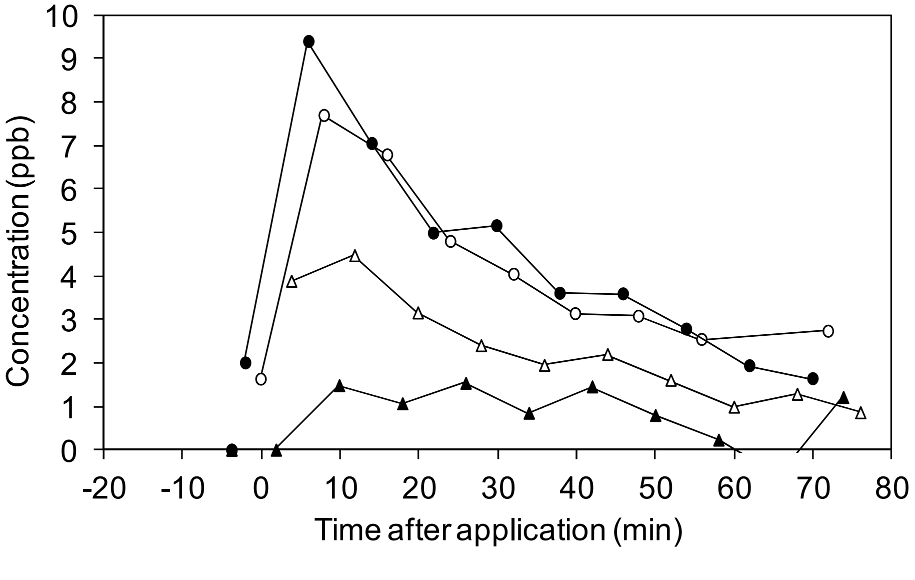

Figure S2. Concentrations of trimethylamine (TMA) in the initial 75 min after application for RAW (○), SEP (△), OZON (●) and SEP-OZON (▲).

1.2. Sulfur Compounds

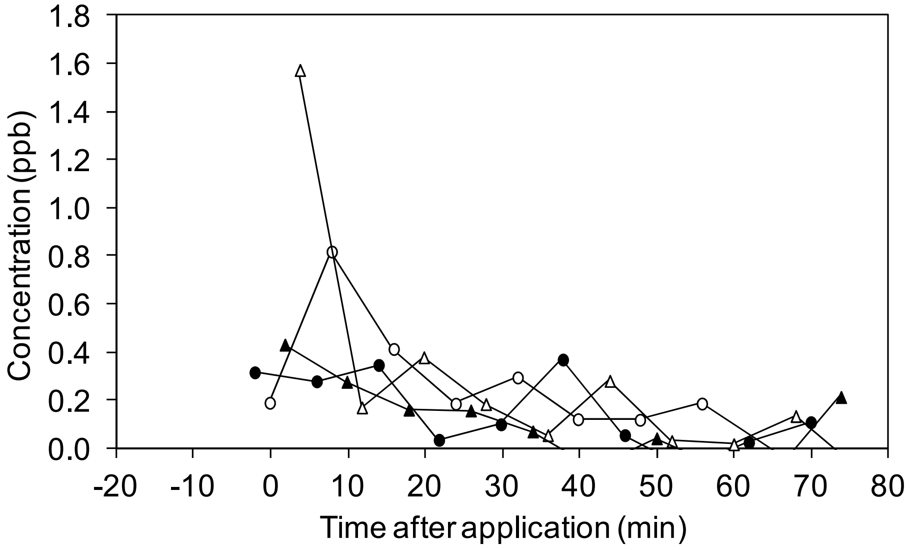

Figure S3. Methanethiol concentration as a function of time for RAW (○), SEP (△), OZON (●) and SEP-OZON (▲).

1.3. Temporal Variation of Carboxylic Acid Emissions

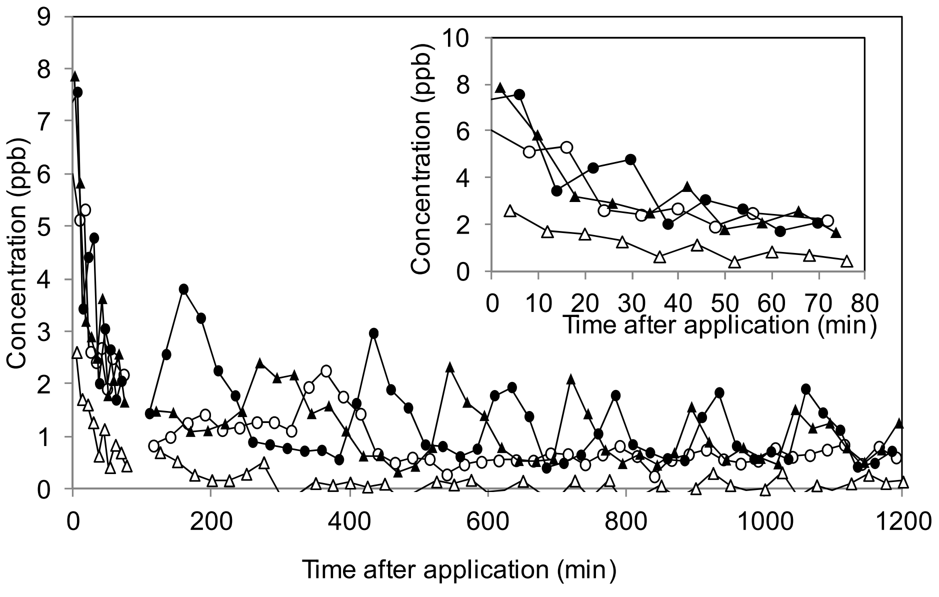

Figure S4. Propanoic acid concentration as a function of time for RAW (○), SEP (△), OZON (●) and SEP-OZON (▲). Insert: Initial 75 min.

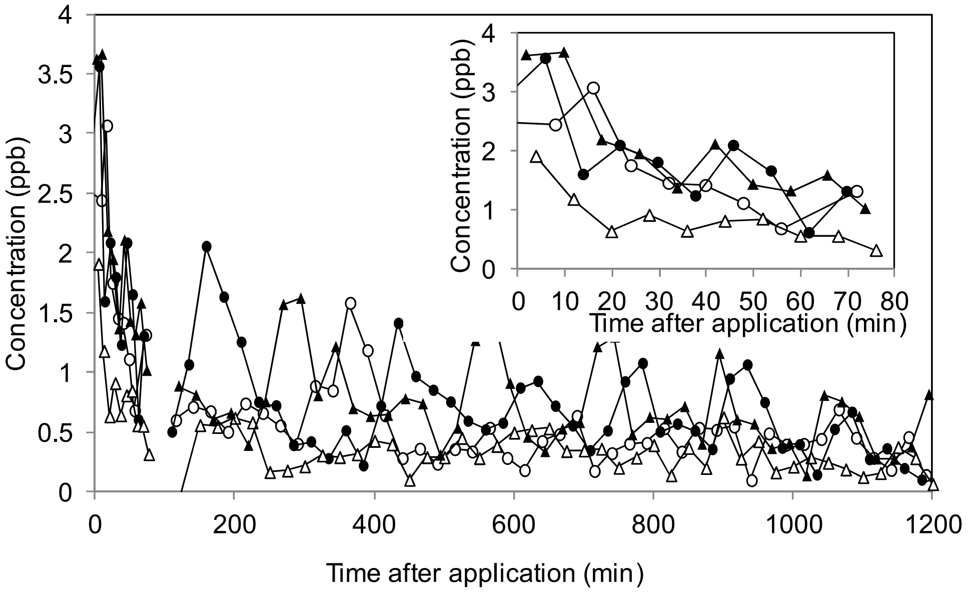

Figure S5. Butanoic acid concentration as a function of time for RAW (○), SEP (△), OZON (●) and SEP-OZON (▲). Insert: Initial 75 min.

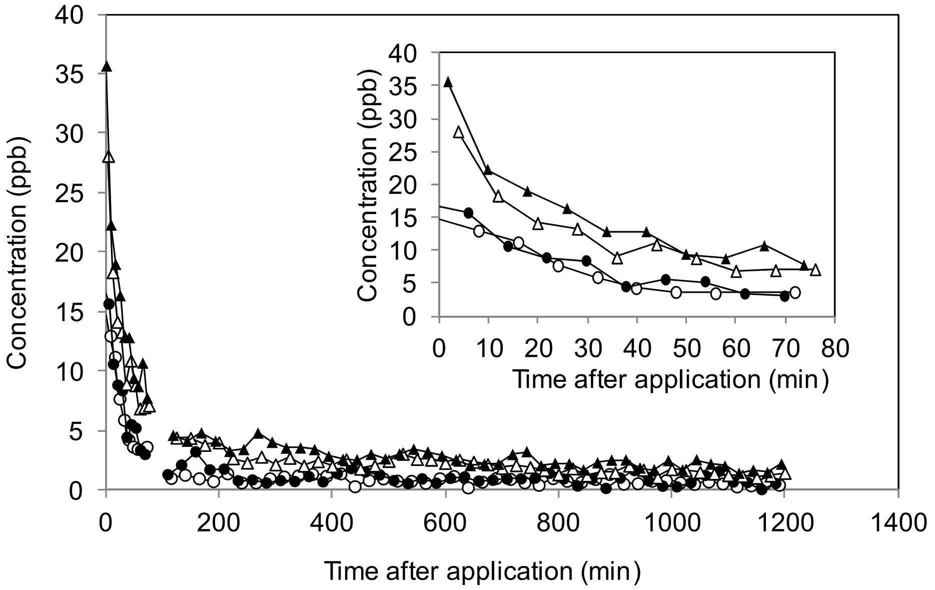

Figure S6. Concentration corresponding to m/z 57 as a function of time. Insert: data from the initial 75 min.

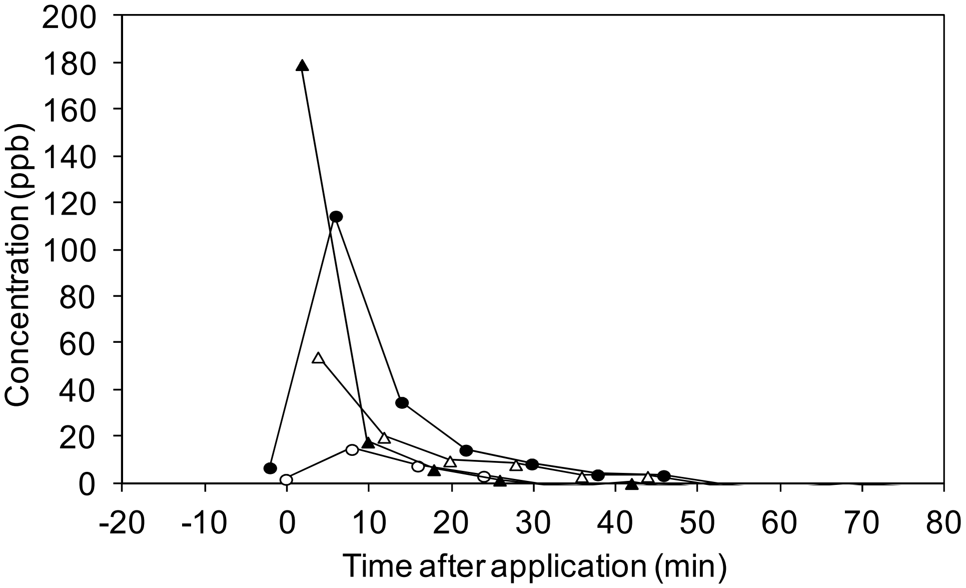

Figure S7. Acetaldehyde (m/z 45) concentration as a function of time for RAW (○), SEP (△), OZON (●) and SEP-OZON (▲).

2. Additional Masses from Mass Scans

Table S2. Masses assigned to aldehydes detected by PTR-MS in full scan mode ~80 min after slurry application. Data is presented as concentration (ppb) assuming a proton transfer rate constant of $2 \times 10^{-9} \cdot cm^3 \cdot molecule^{-1} \cdot s^{-1}$. DL: Detection limit.

Ion with Assignment	OZON	RAW	SEP-OZON	SEP
m/z 69 $C_5H_9^+$; Pentanal fragment ($-H_2O$)	<DL	0.4	1.5	1.5
m/z 83 $C_6H_{11}^+$; Hexanal fragment ($-H_2O$)	<DL	<DL	1.6	<DL
m/z 97 $C_7H_{13}^+$; Heptanal fragment ($-H_2O$)	0.6	0.6	2.4	0.6
m/z 101 $C_6H_{13}O^+$; Hexanal	<DL	<DL	0.7	<DL
m/z 111 $C_8H_{15}^+$; Octanal fragment ($-H_2O$)	0.7	<DL	1.5	<DL
m/z 115 $C_7H_{15}O^+$; Heptanal	<DL	<DL	0.4	<DL

3. TD-GC/MS Data

Odorous compounds are reported as headspace concentration. The data was obtained under dynamic conditions (air flow: 2 L/min) in an acrylic vertical cylinder using a manure volume of 7 L and without stirring. The compounds for which a reference standard was not available (see Table S3 below) are estimated based on the average response factor (= peak area/mass) of the calibrated compounds and these data can thus be regarded as semi-quantitative. Compound identification was based on the NIST library and all peak areas were subtracted by blank values where appropriate.

Table S3. Compounds identified in headspace above slurry by means of TD-GC/MS. Treatments are identical to those used for the soil application experiments in the main article.

Compound	Molecular Weight	Retention Time	Calibration
Acetaldehyde	44	2.10	Relative
Acetone	58	2.46	Relative
Butanal	72	2.82	Relative
Butanone	72	3.01	Relative
2-Methyl-Butanal	58	3.05	Relative
3Methyl-Butanal	57	3.05	Relative
Ethanol	45	3.32	Relative
Pentanal	58	3.94	Relative
2-Ethyl-3-methylbutanal	57	5.66	Relative
Hexanal	72	6.08	Relative
Heptanal	70	8.47	Relative
6-Methyl-2-Heptanone	58	9.46	Relative
Octanal	84	10.41	Relative
Nonanal	98	12.39	Relative
Decanal	82	14.01	Relative
Formic acid	46	14.15	Relative
Benzaldehyde	106	14.47	Relative
2,3-Butanedione	86	3.94	Standard
Acetic acid	60	13.22	Standard
Propanoic acid	74	14.50	Standard
Butanoic acid	60	15.75	Standard
3-Methylthylbutanoic acid	60	16.32	Standard
Pentanoic acid	60	17.17	Standard
Hexanoic acid	60	18.47	Standard
Phenol	94	20.39	Standard
4-Methylphenol	108	21.21	Standard
4-Ethylphenol	107	22.16	Standard
Indole	117	24.92	Standard
3-Methyl-1H-indole	130	25.35	Standard

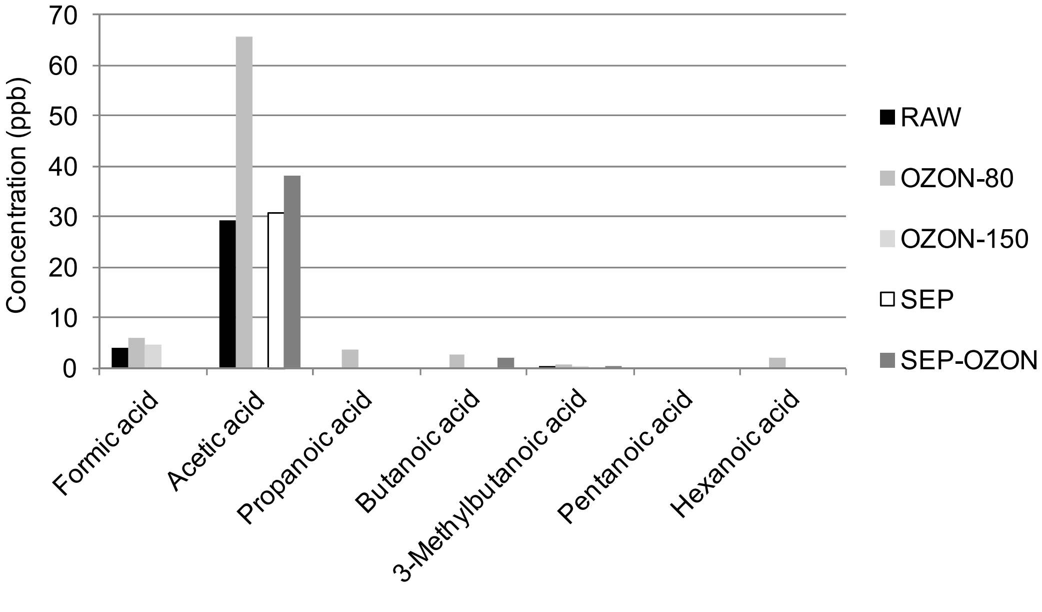

Figure S8. Carboxylic acids measured in headspace above treated/untreated slurry by TD-GC/MS. OZON-80 and OZON-150 indicates data from addition of 80 and 150 mg O_3 per L manure. OZON-80 and OZON-150 indicates data from addition of 80 and 150 mg O_3 per L manure.

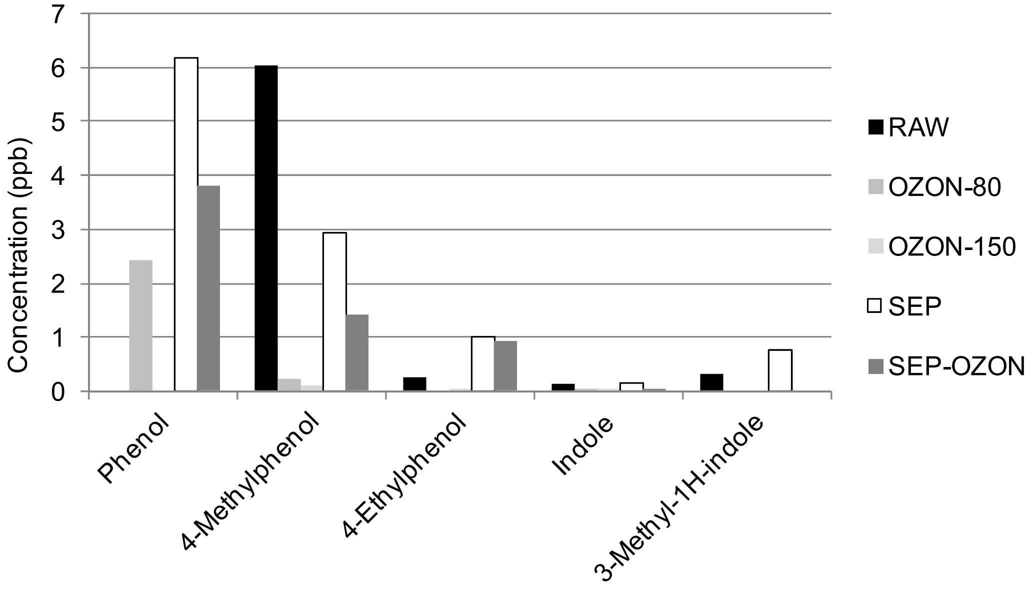

Figure S9. Aromatic compounds (phenols and indoles) measured in headspace above treated/untreated slurry. OZON-80 and OZON-150 indicates data from addition of 80 and 150 mg O_3 per L manure.

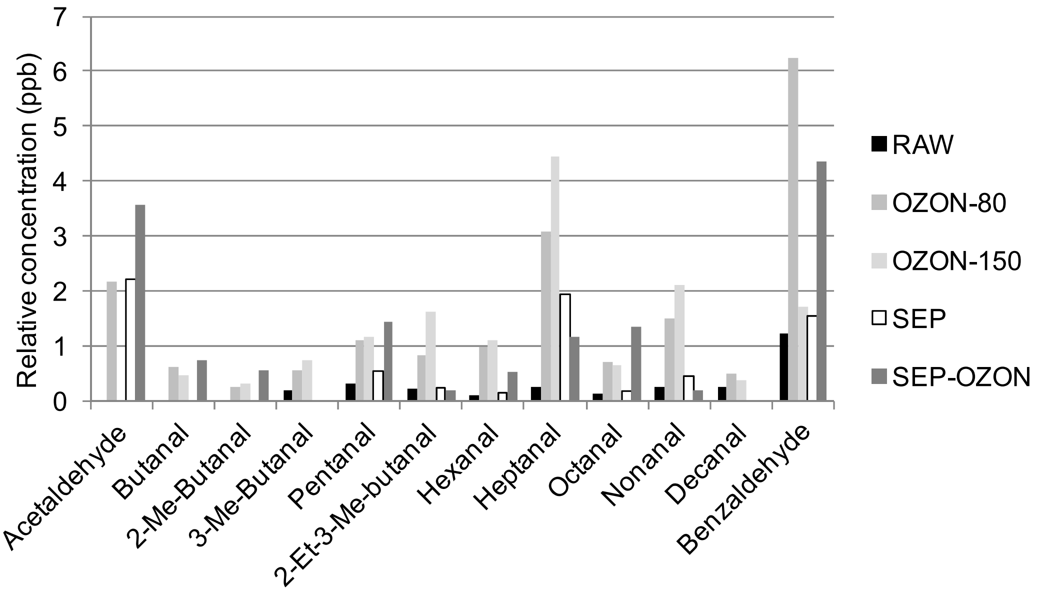

Figure S10. Aldehydes measured in headspace above treated/untreated slurry. The relative concentration was obtained using an average response factor for carboxylic acids and phenols. OZON-80 and OZON-150 indicates data from addition of 80 and 150 mg O_3 per L manure.

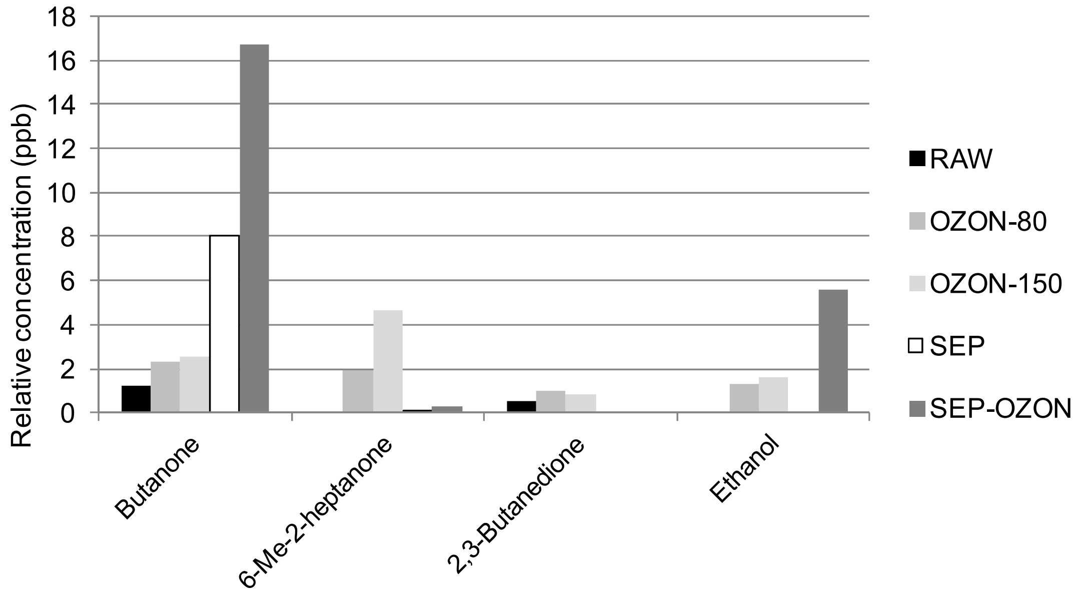

Figure S11. Ketones and ethanol measured in headspace above treated/untreated slurry. The relative concentration was obtained using an average response factor for carboxylic acids and phenols. OZON-80 and OZON-150 indicates data from addition of 80 and 150 mg O_3 per L manure.

4. Comparison of Duplicate Chambers

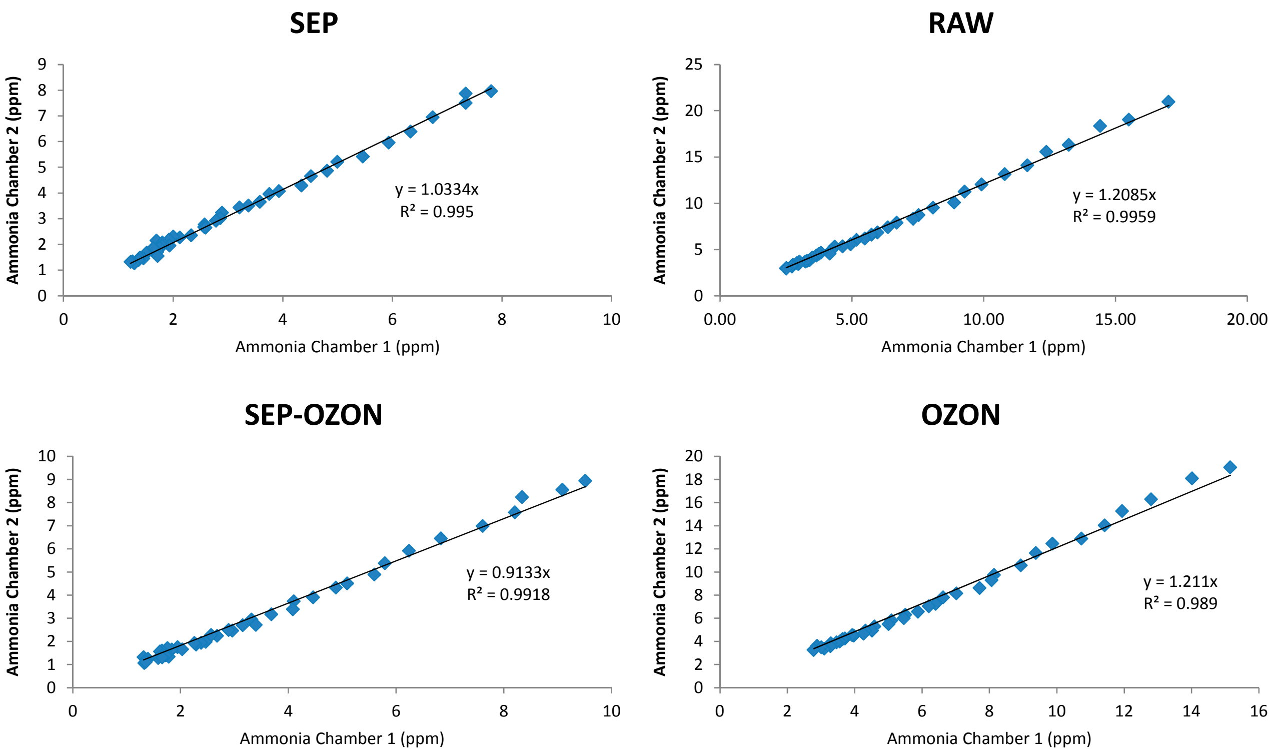

Figure S12. Comparison of duplicated dynamic chambers for the four different slurry treatments with respect to NH_3 concentrations measured by PAD.

© 2015 by the authors; licensee MDPI, Basel, Switzerland. This article is an open access article distributed under the terms and conditions of the Creative Commons Attribution license (http://creativecommons.org/licenses/by/4.0/).
